# The Relation between Red Meat and Whole-Grain Intake and the Colonic Mucosal Barrier: A Cross-Sectional Study

**DOI:** 10.3390/nu12061765

**Published:** 2020-06-12

**Authors:** Mohamad Jawhara, Signe Bek Sørensen, Berit Lilienthal Heitmann, Þórhallur Ingi Halldórsson, Andreas Kristian Pedersen, Vibeke Andersen

**Affiliations:** 1 Focused Research Unit for Molecular Diagnostic and Clinical Research, Institute of Regional Health Research, University Hospital of Southern Denmark- Sonderjylland, 6200 Aabenraa, Denmark; Signe.Bek.Sorensen@rsyd.dk (S.B.S.); va@rsyd.dk (V.A.); 2 Institute of Molecular Medicine, University of Southern Denmark, 5230 Odense, Denmark; 3 Department of Surgery, University Hospital of Southern Denmark-Sonderjylland, 6200 Aabenraa, Denmark; 4 Research Unit for Dietary Studies, the Parker Institute, Bispebjerg and Frederiksberg, 2000 Frederiksberg, Denmark; Berit.Lilienthal.Heitmann@regionh.dk; 5 Section for General Practice, Department of Public Health, University of Copenhagen, 2100 Copenhagen, Denmark; 6 The Boden Institute of Obesity, Nutrition, Exercise & Eating Disorders, The University of Sydney, Sydney, NSW 2006, Australia; 7 Faculty of Food Science and Nutrition, School of Health Sciences, University of Iceland, 101 Reykjavik, Iceland; tih@hi.is; 8 Centre for Fetal Programming, Department of Epidemiology Research, Statens Serum Institut, 2100 Copenhagen, Denmark; 9 Lærings- og Forskningshuset, University Hospital of Southern Denmark, Sonderjylland, 6200 Aabenraa, Denmark; Andreas.Kristian.Pedersen@rsyd.dk; 10 Open Patient Data Explorative Network, University of Southern Jutland, 5230 Odense, Denmark

**Keywords:** colonic mucosal barrier, human colon, large intestine, mucus, mucin, MUC2, red meat, whole-grain

## Abstract

The Colonic Mucosal Barrier (CMB) is the site of interaction between the human body and the colonic microbiota. The mucus is the outer part of the CMB and is considered as the front-line defense of the colon. It separates the host epithelial lining from the colonic content, and it has previously been linked to health and diseases. In this study, we assessed the relationship between red meat and whole-grain intake and (1) the thickness of the colonic mucus (2) the expression of the predominant mucin gene in the human colon (*MUC2*). Patients referred to colonoscopy at the University Hospital of Southern Denmark- Sonderjylland were enrolled between June 2017 and December 2018, and lifestyle data was collected in a cross-sectional study design. Colonic biopsies, blood, urine, and fecal samples were collected. The colonic mucus and bacteria were visualized by immunostaining and fluorescence in situ hybridization techniques. We found a thinner mucus was associated with high red meat intake. Similarly, the results suggested a thinner mucus was associated with high whole-grain intake, albeit to a lesser extent than red meat. This is the first study assessing the association between red meat and whole-grain intake and the colonic mucus in humans. This study is approved by the Danish Ethics Committee (S-20160124) and the Danish Data Protecting Agency (2008-58-035). A study protocol was registered at clinical trials.gov under NCT04235348.

## 1. Introduction

The colonic mucosal barrier (CMB) is the primal site of interaction between the host and the microbiota in the colon and is exposed to enzymes and other physical and chemical compounds in the colonic lumen [1,2,3]. CMB is composed of epithelial cells, isolated from the colonic content by a viscoelastic gel consisting of highly glycosylated mucin proteins, known as the colonic mucus [4,5]. MUC2 is the main protein of the colonic mucus in humans [6]. Through exocytosis, the goblet cells release mucus, which gradually becomes composed of two layers: the inner and the outer mucus [7,8,9]. The firmly-adherent inner mucus acts as a size-exclusion filter for bacteria, and gets converted into a loose, sloppy outer layer as a result of the proteolytic activity of some parasites and bacteria [7,10,11]. The role of the colonic mucus in health and disease has been widely discussed [11,12,13,14]. For instance, structural weakness and permeability of the mucus layer were suggested as important elements in the development of ulcerative colitis [15,16]. The treatment of the impaired mucus was also suggested as a part of the whole treatment of Inflammatory Bowel Diseases (IBD) [17,18,19]. Furthermore, different study protocols have been recently published, assessing the effect of diet on the colonic mucus, and thus on the treatment response on biologics in patients with Chronic Inflammatory Diseases (IBD and rheumatoid arthritis, and other rheumatologic conditions) [20,21]

The relationship between diet and chronic inflammatory diseases has been previously reported, where an increased risk of IBD and rheumatologic conditions was associated with high red meat intake [22,23]. Red meat is composed of many sulfur-containing amino acids (SAAs) and is an essential substrate for sulfide formation by bacteria in the human large intestine (H_2_S) [24]. H_2_S in the colon may reduce the disulfide bonds (S-S) of the mucus, inducing denaturation, and destruction of the architecture of the outer and inner mucus layers [8,25,26]. These mucosal changes may expose the epithelial cells to the luminal contents and may lead to inflammation, epithelial damage, compensatory proliferation, and hyperplasia [27,28,29]. Compared to red meat, the intake of whole-grains was associated with lower risk, and better clinical-course of these diseases [22,30,31]. The bacterial production of short-chain fatty acids from the fermentation of undigested whole-grain fibers may have a protective effect on the colonic mucosa [32,33,34]. Recently, different study protocols were established to assess the effect, and the possible interaction of these dietary components on the clinical-course of IBD, rheumatoid arthritis, and other chronic inflammatory diseases, where an association between the colonic mucus barrier and these dietary components were proposed [20,21,35].

This paper aimed to assess the relationship between intake of red meat and whole-grain and the thickness of the inner mucus layer in biopsy specimens obtained from patients during a colonoscopy in a cross-sectional study design. Furthermore, we assessed the effect of red meat and whole-grain intake on the expression of the main mucus gene (*MUC2*). The study protocol was registered at clinical trials.gov under NCT04235348.

## 2. Materials and Methods

### 2.1. Study Design, Participants, and Setting

This study was described elsewhere [20]. The subjects referred for a colonoscopy at the University Hospital of Southern Denmark—Sonderjylland were invited to participate either by letter to their secure digital mailbox (*n* = 637) [36] or by verbal invitation from the referring general practitioner or the hospital secretaries from the Department of Surgery, Section of Endoscopy (*n* = 24).

Subjects who met the eligibility criteria were prospectively included. The participants had to be adults (≥18 years), and able to read, understand and fill out the questionnaire. Subjects with a history of Inflammatory Bowel Diseases (Crohn’s disease or Ulcerative Colitis), rectum extirpation, left side hemicolectomy, total colectomy, active cancer, previous treatment with biologics, or subjects who have undergone a colonoscopy three months before the inclusion date were excluded. A total number of 161 subjects met the eligibility criteria of this study and were enrolled between 2017 June and 2018 December. These subjects underwent a standard bowel preparation procedure and fasted for 12 h, and were discouraged to eat foods containing visible whole-grains up to five days before the colonoscopy. No restrictions were applied to foods containing milled and pulverized whole-grains, or other dietary fibers. We collected (1) personal data including birthdate and gender; (2) anthropometric data including weight, height for the calculation of the body mass index (BMI); (3) lifestyle and dietary data including red meat and whole-grain consumption; 4) clinical data including indication for the colonoscopy, history of rheumatologic diseases, usage of antibiotics within last 12 months, stool consistency using the Bristol Stool Form Scale [37], and mental and physical health-related quality of life data using the short form 12 item health survey (SF-12) [38]; and biopsy specimens from colon sigmoideum. Colonic biopsies and dietary data were available from 159 subjects and were included in this study. All participants received verbal and written and information and provided verbal and written consent before participation.

### 2.2. Dietary Assessment

The food frequency questionnaire (FFQ) used in this study was developed and validated in relation to the 2007–2008 Danish Health Examination Survey [39]. The FFQ was internet-based and contained information from 267 different food items. The time frame covered by the FFQ was habitual dietary intake during the previous month before bowel preparation for colonoscopy. A photographic food atlas consisting of different food and meal series placed at the end of the questionnaire to quantify the portion sizes for main meals and other main food items. For certain food items such as fruits, whose portion sizes are more standardized, fixed portion sizes were used. The actual weight in grams for each food item was derived by multiplying the reported frequency of consumption with estimated portion sizes. Total energy intake and intake of other nutrients were then quantified using Danish Food Composition Tables [40]. The participants filled out the survey at home using the Research Electronic Data Capture (RedCap) system [41,42], which was sent by an electronic link to their secure digital mailbox [36]. Subjects who were not familiar with the use of computers received technical help from the project nurse. Data was stored in a secure research storage facility [43]. Inspection of the nutritional assessment revealed that two subjects appeared to under-report their energy intake (<2500 KJ/day) and one patient appeared to over-report (>30.000 KJ/day). These outliers (*n* = 3) were managed in two ways: (1) The red meat and whole-grain intake of these outliers were estimated using multiple imputations based on data from other observations (*n* = 156), and (2) sensitivity analyses were conducted omitting these outliers (*n* = 3).

### 2.3. Assessment of the Mucus Thickness

#### 2.3.1. Selected Population and Sample Size Calculation

**Figure 1:**
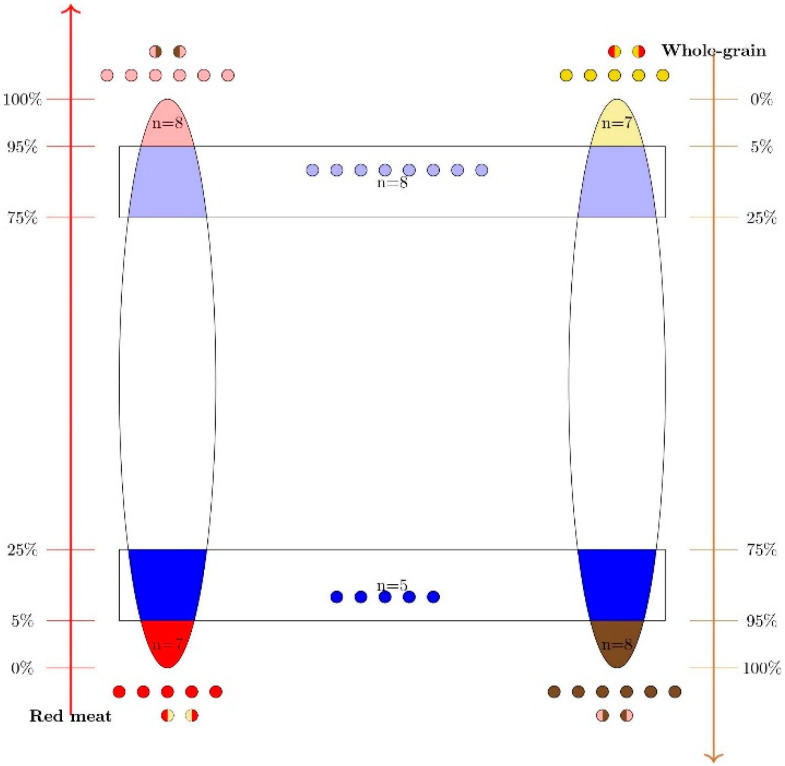
This figure represents the methods used in the selection of participants for assessment of mucus thickness in biopsies from colon sigmoideum based on their reported dietary intake of red meat and whole-grain. The two left and right major ellipsoids represent the 159 recruited subjects, ordered after the reported intake of red meat and whole-grain, respectively. Note that the two axes representing the proportions of the sample at the left and right side of the figure are in opposite directions. The pink and brown areas represent the upper 5th percentiles of the sample with respect to red meat and whole-grain intake, respectively. The red and yellow areas represent the lower 5th percentiles of the sample, with respect to red meat and whole-grain intake, respectively. The circles illustrate subjects found uniquely in one 5th percentile. The two-colored circles illustrate four subjects found in more than one 5th percentile simultaneously (eight two-colored circles). Furthermore, the upper rectangle included subjects located simultaneously at the upper red meat and lower whole-grain quartiles (*n* = 8). The lower rectangle included subjects located simultaneously at the lower red meat and upper whole-grain quartiles (*n* = 5). The sum of these subjects (*n* = 39 was selected as the targeted study population.

The analysis of the association between red meat and whole-grain intake and the mucus thickness was evaluated in 39 subjects. This selection was based on the reported dietary intake of red meat and whole-grain as described in the following model and illustrated in Figure 1.

- The upper 5th percentile of the sample (*n* = 8) with respect to red meat intake (marked in pink in Figure 1)
- The lower 5th percentile of the sample (*n* = 7) with respect to red meat intake (marked in red in Figure 1)
- The upper 5th percentile of the sample (*n* = 8) with respect to whole-grain intake (marked in brown in Figure 1)
- The lower 5th percentile of the sample (*n* = 7) with respect to whole-grain intake (marked in yellow in Figure 1)
- Observations located in the lower red meat quantile and simultaneously in the upper whole-grain quantile (*n* = 5) (marked in blue in Figure 1)
- Observations located in the upper red meat quantile, and lower whole-grain quantile (*n* = 8) (marked in light blue in Figure 1)

This model revealed a total of 43 observations. Some of these were identified in more than one group resulting in *n* = 39 subjects (the two-colored circles in Figure 1).

In the calculation of the sample size needed for the analyses between diet and mucus thickness, we performed a power calculation using a Monte Carlo simulation [44], based on our knowledge from other papers with the statistical software Stata 15 [45]. A sample size of 20 subjects was deemed adequate to achieve a power of 80% with α of 0.05 and β value of 0.8 using a mixed effect model.

#### 2.3.2. Cryosectioning and Fixation

Colonic specimens were obtained from endoscopically normal appearance mucosa of the colon sigmoideum, 25–35 cm from the anus using 2.8 mm biopsy forceps, immediately snap-frozen in liquid nitrogen, and stored at −80 °C until sectioning. Frozen samples were then emerged in Optimal Cutting Temperature (OCT) compound and sectioned to 8 μm thickness at −25 °C using an NX50 cryostat (HM 505N Cryostar, Thermo Scientific, Walldorf, Germany) and placed on FLEX IHC Microscope Slides (Dako, Glostrup, Denmark # K8020). Slides were placed to thaw for 1 h at 4 °C [46]. Water-free methanol was used rather than ethanol to preserve the intestinal mucus as ethanol was previously reported to disrupt the mucus [47]. The colonic sections were fixed in methanol-Carnoy’s fixative (60% dry methanol, 30% chloroform, 10% glacial acetic acid) for 30 min at 4 °C, washed in PBS thrice for 10 min. The sections were then washed in 99% ethanol thrice for 10 min, air-dried for 10–15 min, and then directly (1) fluorescence in situ hybridized (FISH) and (2) immunostained. FISH was performed before mucus staining.

#### 2.3.3. Fluorescence In Situ Hybridization

FISH targeting bacterial cells was performed using the oligonucleotide probe Eub338 [48] (5′-GCT GCC TCC CGT AGG AGT-3′) custom synthesized and 5′ labelled with the fluorophore Cy3 (extinction’s wavelength, 552 nm; emission wavelength, 570 nm). The labelled probe was obtained from Eurofins Genomics (Ebersberg, Germany). The method has been previously described [49]. The sections on microscope slides were incubated overnight in a solution of 50 °C pre-warmed 100 µL hybridization buffer of 0.9 M NaCl, 0.02 M Tris-HCl pH 7.5, 0.1% SDS mixed with 1 µg probe (2 μL 500 ng/µL probe), and applied to the hybridization chamber’s top. The lid was placed on the Shandon rack, wrapped in tinfoil, and incubated overnight at 50 °C. The slides were then removed from the cover plate and placed in a Coplin jar with 200 mL washing buffer (0.9 M NaCl, 0.02 M Tris pH 7.5) preheated to 50 °C and left for 20 min at 50 °C, and then washed in PBS thrice for 10 min.

#### 2.3.4. Immunostaining and DAPI Fluorescent Staining

Sections on microscope slides were treated with a blocking solution (5% Fetal Bovine Serum in PBS), and the slides were incubated in a dark and humid environment for 30 min at 4 °C. The blocking solution was then removed and substituted by 200 µL of a 1:50 solution of Anti-MUC2 antibody (Ccp58) (ab118964) diluted in blocking solution. The slides were incubated for 22 h at 4 °C in a dark and humid environment. After incubation, the sections were washed in PBS 3x for 10 min Slides were then treated with 200 µL of a 1:1500 solution of the Goat Anti-Mouse IgG H&L (Alexa Fluor® 488, ab150113, Abcam, Cambridge, United Kingdom) diluted in blocking solution and incubated for 2 h at 4 °C in a dark and humid environment. The slides were then washed in PBS thrice for ten minutes, dipped in distilled water, drained and air-dried.

The sections were mounted using Vectashield with DAPI (H-1200) (Vector Laboratories, Inc. Burlingame, CA 94010, USA). Sections were allowed to sit at room temperature for 30 min and then preserved at −20 °C for two weeks before imaging.

#### 2.3.5. Mucus Visualization and Assessment

The software NDP.view2 was used to measure the thickness of the brightly stained region located just interior to the epithelium [50]. The assessors were blinded for other data. The thickness was measured perpendicularly to the epithelial tissue at three to five different points at which independent measurements can be obtained. The measurements were omitted at locations where folding of the section made measurement impossible or where large breaks occurred between the mucus.

### 2.4. Quantitative Real-Time PCR of MUC2 Expression in Colonic Biopsies

Colonic specimens were obtained from endoscopically normal appearance mucosa of the colon sigmoideum, 25–35 cm from the anus using 2.8 mm biopsy forceps, stained in RNAlater solution for 24 h and stored at −80 °C. Total RNA was isolated using TRIzol reagent (Thermo Fischer Scientific) according to the manufacturer’s instructions. The RNA concentration and purity were measured using NanoDrop (Thermo Scientific, Wilmington, DE, USA). Complementary DNA (cDNA) was transcribed from isolated RNA using the M-MLV Reverse Transcriptase kit (Sigma-Aldrich, Darmstadt, Germany) according to the manufacturer’s instructions. Quantitative Real-time PCR was performed in technical triplicates on a StepOnePlus Real-Time PCR system (Life Technologies, Carlsbad, CA, USA) using TaqMan Universal Mastermix II (Applied Biosystems, Foster City, CA, USA) and certified Gene Expression Assays. Gene expression of *MUC2* (Hs03005094) was normalized as n-fold difference to EPCAM (Hs00158980) according to the cycling threshold. Calculation of mRNA levels was performed with the StepOne Software (Version 2.3., Thermo Fisher, CA, USA).

### 2.5. Statistical Analysis

Descriptive statistics were computed. Frequencies were calculated for categorical data. Continuous data was presented as means with standard deviation (SD) or medians with interquartile range (IQR) depending on the distribution of the data. The age (year) was calculated at the inclusion date. Nutrients data were not adjusted to the total energy intake as the assessment of gross intake of red meat and whole-grain is relevant where the colonic mucus is directly exposed to these nutrients or their products of digestion. SF-12 data was analyzed and SF-12 s-version t-scores were calculated using a Stata module [51].

To avoid information bias, multiple imputations were applied to estimate red meat and whole-grain intake as three subjects (2%) of the whole study population or 8% of the sample selected for mucus thickness evaluation appeared to underreport (<2500 KJ) or over-report (>30.000 KJ) energy intake. Multivariate normal imputation was applied with 100 imputed datasets and dietary fibers, fat, age, and sex as independent variables. Bowel transit time was considered as mediator and was hence not controlled for in the data analyses.

A mixed-effect linear regression model was used to analyze the mucus thickness (continuous, log-transformed) outcomes. The model followed a three-step approach: (1) a partially-adjusted model assessing the association between each exposure variable and the thickness of the colonic mucus; (2) a multivariate model where the two exposure variables were included in the same model; and (3) a sensitivity analysis where the three subjects with outlier values from analyses were excluded. To avoid overfitting of the models, the one-in-ten rule was followed for fixed effects and the one-in-twenty rule for random effects. The analyses were adjusted for potential confounders including age, gender, and BMI. The coefficients and confidence intervals were converted to percentage using the following formula (exp(β)-1)⋅100.

Gamma regression analyses were applied to evaluate the impact of red meat and whole-grain intake on the expression of MUC2 gene in biopsies. All statistical analyses were carried out using the statistical software “Stata 15” [45]. The Stata output of all analyses is presented in Supplementary Appendix.

### 2.6. Ethical Considerations

All participants in this study gave written informed consent before participation. This study is approved by the Danish Ethics Committee (S-20160124) and the Danish Data Protecting Agency (2008-58-035).

## 3. Results

### 3.1. The Main Characteristics of the Study Population

The main clinical and demographic characteristics of the study population (*n* = 159) are presented in Table 1. Normally distributed continuous data are presented as means and standard deviations, and skewed data are presented as medians and interquartile ranges unless stated otherwise.

The study population had a slight predominance of men (57%). The age of the subjects ranged from 26 to 83 years; 14% of them were active smokers. The major indication for the colonoscopy was a control after previous polypectomy (44.7%) followed by a changed stool pattern (14%) and a familial disposition to colorectal cancer (14.5%). BMI ranged between 17.3 and 52.2 kg/m^2^. The mental and physical health-related quality of life data of the included subjects using SF-12 is presented in Table S1. In general, subjects with good physical and mental health were predominant. The dietary intake data based on the 30-day food frequency questionnaire were not normally distributed and were presented as median and interquartile range (IQR) in Table 2.

### 3.2. The Colonic Mucus Thickness

**Figure 2:**
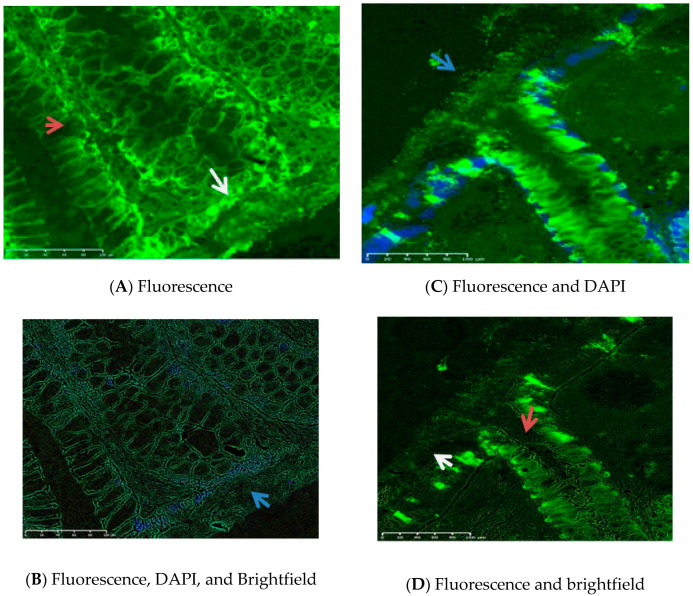
MUC2 staining of colonic mucosa. These images represent the immuno- and DAPI fluorescent stained mucus of the colon sigmoideum from two different subjects. Images (**A**,**B**) are from subject 1, and (**C**,**D**) are from subject 2. The mucus is stained in green using Anti-MUC2 antibody (Ccp58) (ab118964) in all images. In subject 1: Image (**A**) represents the fluorescence immunostaining of the mucus (green). The white arrow indicates the inner mucus from the apical membrane side. The red arrow is located in a colonic crypt and indicates the colonic goblet cells; Image (**B**) represents the fluorescent immunostaining of the mucus (green), DAPI staining of mononuclear cells (blue), where the Brightfield filter was applied. The blue arrow indicates the luminal side of the inner mucus surface. In subject 2, image (**C**) represents the fluorescence immunostaining of the mucus (green) and DAPI staining of mononuclear cells (blue). The blue arrow indicates the luminal side of the inner mucus surface; Image (**D**) represents the fluorescent immunostaining of the mucus (green) where the Brightfield filter was applied. The white arrow indicates the inner mucus from the apical membrane side. The red arrow is located in a colonic crypt and indicates the colonic goblet cells. Scale bar: 100 μm in all images.

The superficial mucus layer was absent from the surface of all biopsies. Few bacterial strains were spread over the slides instead of a regular bacterial layer habiting the outer and covering the inner mucus. The inner mucus layer was preserved in all biopsies and was slightly detached from the apical membrane. The mean (SD) mucus thickness was 19.4 (9.9) µm and ranged between 6.2 and 45.6 µm. Epithelial defects were not detected. The mucus thickness of the included sample (*n* = 39) is presented in Table 3. Images of the mucosa are presented in Figure 2. Supplementary images presenting bacterial strains in samples are presented in Figure S1.

#### 3.2.1. The Association between Red Meat, Whole-Grain Intake and Colonic Mucus Thickness

The association between red meat, whole-grain intake, and the mucus thickness was assessed as univariate and multivariate analyses (*n* = 39) based on multiple imputations. Supplementary sensitivity analyses were applied, where the three subjects with outlying data (energy intake < 25 MJ or > 30 MJ) were omitted (*n* = 36). The regression coefficients, *p*-values, and 95% confidence intervals are presented in Table 4. The assessment of age, gender, and BMI as potential confounders was presented in a hierarchical framework.

Red meat and whole-grain intake were associated with a thinner mucus. In partially adjusted analyses, where the association of mucus thickness with red meat was assessed separately from whole-grain intake, the mucus was 17% (*p* = 0.02) thinner for each 100 g per day higher red meat intake in non-adjusted analysis and up to 19% (p = 0.016) thinner after adjustment for age and gender. BMI data was available for (*n* = 36) subjects. When adjusting for BMI the association was attenuated with the mucus thickness now being 13% (*p* = 0.12) to 11% (*p* = 0.16) thinner for each 100 g higher intake per day, but the difference became non-significant. When whole-grain intake was combined in an adjusted multivariate test, the results became non-significant in the majority of the analyses. The mucus was 15% (*p* = 0.034) thinner for each 100 g higher red meat intake per day when adjusted for age and gender. Similar coefficients were observed in the sensitivity analysis.

A high whole-grain intake was significantly associated with a thinner mucus layer in both univariate and multivariate analyses, even after adjustment for age, gender, and BMI. The mucus was 9 to 12% thinner for each 100 g higher whole-grain intake/day. Similar results were observed in the sensitivity analyses.

#### 3.2.2. The Association between Red Meat, Whole-Grain Intake and MUC2 Gene Expression

Colonic biopsies were available in 157 subjects. *MUC2* expression was assessed in 154 subjects after the three outliers were omitted (*n* = 154). No association was found between red meat and whole-grain intake and the expression rate of *MUC2*. The regression coefficients, *p*-values, and 95% confidence intervals are presented in Table 5.

## 4. Discussion

To our knowledge, this is the first study to explore the association between intake of certain components of the diet and the colonic mucus thickness in humans. Our results suggested that high red meat intake was associated with a thinner inner mucus layer in humans and that high whole-grain intake, albeit to a lesser degree than red meat, was associated with a thinner mucus layer. No associations were found between the expression of the main colonic mucin (*MUC2*) and red meat or whole-grain intake.

High red meat intake has been associated with an increased risk of developing chronic inflammatory diseases and colorectal cancer [23,53]. Red meat intake has been previously suggested to harm the colonic mucus in different studies on rodents [21,26]. The microbial formation of H2S from the digestion of red meat was suggested to bind to disulfide bonds holding MUC2 molecules together, and thus to contribute to a destruction of the mucus architecture [26,54,55]. The results of this study showed a 10 to 19% thinner inner mucus associated with 100 g higher red meat intake per day in humans, and the regression coefficients were stable in partially adjusted analyses. The associations became stronger after adjusting for age and gender, but non-significant when adjusted for BMI and whole-grain intake. As expected, gender and age seemed to have an effect on the colonic mucus thickness. Previous studies have suggested differences in the length of the rectosigmoid colon in humans, where women were observed to have a longer rectosigmoid colon, and thus a larger mucosal surface, which may support the results [56,57]. Age was also previously reported as a factor contributing to thinner mucus in mice [58]. When adjusted for BMI, the associations became statistically non-significant. Different factors may contribute to these results. BMI data were only available for 36/39 subjects in the main analyses and 35/39 in the sensitivity analyses, which could have reduced the statistical power. Another explanation could be that BMI was associated with the mucus thickness. Subjects with high BMI may have a larger mucosal surface, which may reduce the association between red meat, and H_2_S concentration and a particular part of the mucosa. However, this explanation may be more justified in the gastric mucosa, and less justified in the colonic mucosa. Different factors could have an impact on the mucosal surface, like gender, obstipation, and diverticulosis [56,57]. Another explanation could be that reporting bias of dietary data was highest among subjects with high BMI. It was not possible to include BMI as an independent variable in multiple imputation data, as BMI data was missing in two of the three outliers. Other studies are needed to explore whether BMI is associated with mucus thickness before strong conclusions can be drawn.

The consumption of whole-grains and dietary fiber is deemed to have a health-beneficial effect compared to red meat [34,59]. The inner mucus layer was 9 to 12% thinner for each 100 g higher whole-grain intake per day, and these results are in line with previous findings on animal studies [60].Meldrum et al. demonstrated the enhancement of intestinal porcine mucin (MUC2) binding to plant cell wall structures from fruit and grain mediated by soluble dietary fibers embedded within cellulose networks mucin [60]. With microscopic measurements, the authors showed that neutral dietary fiber polysaccharides are capable of disrupting the mucin network, which facilitates the interpenetration of mucin molecules into the polysaccharide mesh, and thus influencing the mucus barrier properties. Intake of red meat and whole-grain may act in synergy, and we, therefore, ran a supplementary sensitivity analysis to test for interactions between red meat and whole-grain in relation to colonic mucus thickness by introducing a product term (red meat x whole grain) in the regression models but found no evidence of interaction effects (*p* = 0.569).

The inner mucus layer was preserved in all samples (*n* = 39), while the superficial mucus layer was absent in all samples. Few bacterial strains were spread over the slides instead of a regular bacterial layer habiting the outer and covering the inner mucus. Similar observations were previously reported as a consequence of the bowel preparation procedure before the colonoscopic investigation. In an RCT, Bucher et al. analyzed the effect of bowel preparation on the morphologic alterations of the colonic mucosa and reported a loss of the superficial mucus in 96% of subjects undergoing a mechanical bowel preparation [61]. Similar observations were also described in animal studies [62]. The outer layer, which is the habitat of commensal bacteria in the colon, is loosely attached and may be easily removed, even by a simple suction, whereas the inner layer is tightly attached to the apical membrane [63,64]. Although these changes to the superficial mucus layer were observed, we assume that the loss of the outer mucus does not influence the results, as they targeted the inner mucus layer, which was preserved.

The mean (SD) thickness of the inner mucus layer in this study was 19 (10) µm, which is considerably lower compared to other studies. The absolute thickness measured in individual studies may depend on the study population, sample processing, and measurement technique, which may have contributed to the variability of the measurements [47,65]. Therefore, it is difficult to compare the results of this study to results from previous studies that examined mucus thickness, due to the preparation used for colonoscopy and sample-processing techniques, both of which could have influenced the results. The bowel transit time is an important factor that may influence the colonic mucus. Fiber has been shown to increase gut motility, which may impact mucus thickness [66]. Bowel transit time was considered in our analyses as mediator in the relationship between the exposure and the outcome, and was hence not controlled for in the data analyses [67,68].

Our data showed no association between the expression of *MUC2* in the colonic specimens and the intake of red meat or whole-grains (*n* = 154). Paturi et al. assessed the influence of three prebiotics: (1) fructo-oligosaccharide, (2) galacto-oligosaccharide, and (3) inulin on the expression of *MUC2* in rats. No difference was found between the intervention and control rats [69]. To our knowledge, no available studies have assessed the effect of red meat on *MUC2* expression. Replication of the findings from this study in independent data is required before strong conclusions can be drawn.

This is the first study assessing the association between red meat and whole-grain intake and the colonic mucus in humans, where the potential effect of diet is generally more complex to study compared to animal studies. This study has many strengths. The assessors were blinded, which reduces the risk of detection bias. The application of multiple imputations in outliers may reduce the risk of attribution bias. The subjects included in this study were enrolled in Denmark, where the consumption of whole-grain products is relatively high, compared to other regions of the world [70]. Moreover, the samples assessed consisted of 39 subjects with an extremely low or high intake of red meat and whole-grains. This population allowed the assessment of the relationship between high exposure on the colonic mucosa compared to low exposure and enables us to observe possible disadvantages from high whole-grain intake. However, there are several limitations that need acknowledgement. The FFQ was validated and found to be valid for application in our study population, but had a recall period of 30 days, which may impact the reliability of the nutritional data. Any biomarkers with better prediction of whole-grain or red meat medium to long-term intake compared to conventional methods of reporting did not exist at the time of writing [71,72]. Another limitation is that the biopsy specimens were obtained after a bowel preparation procedure, which removed the outer mucus layer. However, these changes resulting in the removal of the loose mucus, allowed a more precise measurement of the functional inner layer. Another limitation is that the colonic specimens were obtained in patients discouraged to eat foods containing visible whole-grains up to five days before the colonoscopy, as standard instruction of the procedure, without any restrictions to foods containing milled and pulverized whole-grains, or other dietary fibers. However, a change in this instruction would have resulted in a delay of the procedure, discomfort for the patient and malfunction of the scope. Drawing conclusions on mucus bacterial induced degradation of the colonic mucus under these conditions would be extremely challenging.

In conclusion, we explored the association between red meat and whole-grain intake and the thickness of the colonic mucus, which is considered the front-line defense of the colonic mucosal barrier in humans. Our results suggested that subjects with high red meat intake might have a thinner inner mucus in the colon. Similar results were observed for whole-grain intake. A positive health-related effect of dietary fiber and whole-grain consumption is well acknowledged, and many national dietary guidelines promote whole-grain consumption. However, this study also reports a potential disadvantage of high whole-grain consumption on the colonic mucus barrier. No associations were found between red meat or whole-grain intake and *MUC2* expression in colonic biopsies. A better understanding of the biological background of the effect and the consequences of red meat and whole-grain intake on the colonic mucus is needed in future studies.

## Figures and Tables

**Table 1:** Demographic and clinical characteristics of the study population (*n* = 159), and the sample used for the analyses of the relation between diet and mucus thickness (*n* = 39).

Characteristics		Study Population (*n* = 159)	Selected Subjects (*n* = 39)
Gender	Men	90 (57%)	23 (59%)
	Women	69 (43%)	16 (41%)
Age (year)	mean (SD)	66 (10)	65 (11)
Anthropometrics	Weight (kg), mean (SD)	83 (16)	84 (13)
	Height (cm), mean (SD)	173 (9)	174 (9)
	Body mass index (kg/m^2^), median (IQR)	28 (25, 30) *	28 (25, 29) ^#^
Smoking status	Daily	18 (11%)	4 (10%)
	Occasionally ^1^	4 (3%)	1 (3%)
	Former smokers ^2^	76 (48%)	21 (54%)
	Non-smokers ^3^	61 (38%)	13 (33%)
Accidental weight loss within the last 3 months		11 (7%)	2 (5%)
Bristol Stool consistency ^4^	Type 1	5 (3%)	1 (3%)
	Type 2	12 (8%)	3 (9%)
	Type 3	38 (24%)	12 (34%)
	Type 4	67 (42%)	13 (37%)
	Type 5	8 (5%)	2 (6%)
	Type 6	10 (6%)	3 (9%)
	Type 7	10 (6%)	1 (3%)
	Missing values	9 (6%)	4 (10%)
Indication of colonoscopy	Adenoma control	71 (45%)	16 (41%)
	Changed stool pattern	23 (15%)	8 (21%)
	CRC disposition ^5^	23 (15%)	9 (23%)
	Hematochezia	23 (14%)	5 (13%)
	Post-operative control	14 (8%)	1 (3%)
	Other indications	5 (2%)	-
Main findings from colonoscopy	Colon diverticulosis	50 (31%)	12 (31%)
	Adenomas	48 (30%)	9 (23%)
	Normal colon	47 (30%)	13 (33%)
	Insufficient bowel preparation	5 (3%)	-
	Other findings ^6^	13 (8%)	5 (13%)
History of Antibiotics	Used during the last 2 weeks ^7^	6 (4%)	-
	Used during the last 2 months ^7^	16 (10%)	3 (8%)
	Used during last 12 months ^7^	36 (23%)	8 (21%)

The demographic and clinical characteristics of the study population (*n* = 159) and the selected subjects for mucus thickness assessment (*n* = 39). ^1^ Less than one event/day; ^2^ Smoking cessation before the inclusion date; ^3^ As active smoker; ^4^ Bristol Stool Form Chart Scoring System with categorical scores ranging from one to seven, where type one corresponds to a hard stool, and type seven to an entirely liquid stool [52]; ^5^ Familial disposition to colorectal cancer; ^6^ other findings such as cancer, acute diverticulitis, hemorrhoids, etc.; ^7^ Patient reported outcome. * Missing values in 15/159; ^#^ Missing values in 3/39. Values are rounded to whole numbers.

**Table 2:** The dietary intake of the study population (*n* = 156) ^1^ and the selected subjects for assessment of mucus thickness (*n* = 36) ^1^.

Daily Total Energy and Dietary Intake	Study Population ^1^ (*n* = 156)	Selected Subjects ^1^ (*n* = 36)
	Median (IQR) ^2^	Median (IQR) ^2^
Total energy (MJ)	8.5 (7.0, 10.6)	9.5 (8.1, 12.5)
Red meat (g)	76 (55, 112)	114 (46.7, 162.7)
Whole-grains (g)	143 (112, 192)	118.6 (42.4, 300.3)
Fat (g)	29 (19, 40)	28.6 (16.1, 37.4)
Dietary fibers (g)	23 (18, 30)	24.4 (16.0, 39.3)

The 30-day dietary intake based on data from food frequency questionnaire of the total study population (*n* = 156) and the selected subjects for the assessment of associations between red meat and whole-grain and the colonic mucus thickness (*n* = 36). ^1^ Three outliers were not included in this table; ^2^ Interquartile range. Values for total energy intake are rounded up to the nearest first decimal place. Other values are rounded to the nearest whole number.

**Table 3:** The individual mean of the mucus thickness in 39 subjects.

Mucus Thickness	Number of Subjects (%)
<10 µm	5 (13)
11–20 (µm)	17 (44)
21–30 (µm)	11 (28)
>30 (µm)	6 (15)

The mean of the mucus thickness in biopsies from colon sigmoideum was measured three to five times per biopsy.

**Table 4:** The change in mucus thickness in biopsies from colon sigmoideum (percent) per 100 g difference in red meat or whole-grain intake.

Exposure Variables	Partially Adjusted Model ^1^ (*n* = 39)		Adjusted Multivariate Analyses ^2^ (*n* = 39)		Multivariate Sensitivity Analyses ^3^ (*n* = 36)	
	Percent (95% CI)	*p*-Value	Percent (95% CI)	*p*-Value	Percent (95% CI)	*p*-Value
Non-adjusted for potential confounders						
Red meat	−17 (−29, −2)	0.024	−13 (−25, 1)	0.065	−13 (−25, 2)	0.089
Whole-grains	−12 (−18, −4)	0.002	−10 (−17, −3)	0.007	−9 (−16, −2)	0.019
Adjusted for age						
Red meat	−19 (−31, −5)	0.008	−16 (−27, −2)	0.022	−15 (−27, −1)	0.035
Whole-grains	−12 (−19, −5)	0.001	−10 (−17, −3)	0.004	−10 (−16, −2)	0.012
Adjusted for age and gender						
Red meat	−18 (−30, −3)	0.017	−15 (−27, −1)	0.034	−14 (−26, 1)	0.062
Whole-grains	−11 (−18, −4)	0.002	−10 (−17, −3)	0.005	−9 (−16, −2)	0.014
Adjusted for BMI						
Red meat	−13 (−26, 4)^+^	0.122	−11 (−24, 4) ^+^	0.143	−11 (−24, 4) ^++^	0.157
Whole-grains	−11 (−18, −4)^+^	0.003	−11 (−17, −4) ^+^	0.004	−10 (−17, −3) ^++^	0.008
Adjusted for age, gender, and BMI						
Red meat	−11 (−24, 5) ^+^	0.163	−10 (−22, 4) ^+^	0.159	−10 (−22, 4) ^++^	0.162
Whole-grains	−10 (−16, −3) ^+^	0.005	−9 (−16, −3) ^+^	0.005	−10 (−16, −3) ^++^	0.006

The change in mucus thickness in biopsies from colon sigmoideum (percent) per 100 g difference in red meat or whole-grain intake was conducted in: ^1^ Partially adjusted model where red meat and whole-grain intake were assessed independently in relation to the colonic mucus thickness using Multiple-imputation estimates linear mixed-effect models (*n* = 39); ^2^ a multivariate model where the two exposure variables where included in the same model using Multiple-imputation estimates multivariate linear mixed-effect models (*n* = 39); and ^3^ a sensitivity analysis where we excluded the three subjects with outlier values from analyses using multivariate linear mixed-effect models (*n* = 36). ^+^ Body mass index (BMI) data were available in (*n* = 36); ^++^ BMI data were available in (*n* = 35); Abbreviations: BMI, Body mass index; CI, confidence interval.

**Table 5:** The association between red meat and whole-grain intake and *MUC2* expression data (*n* = 154).

Variables	Coefficient (95% CI)	*p*-Value
Red meat	1.09 (−0.93, 1.27)	0.280
Whole-grain	1.06 (0.90, 1.24)	0.468

The association between the intake of red meat and whole-grain intake with *MUC2* expression in biopsies from colon sigmoideum in univariate gamma-regression analyses.

## Supplementary Materials

The following are available online at https://www.mdpi.com/2072-6643/12/6/1765/s1, Table S1: The mental and physical health-related quality of life data of the included subjects using SF-12 questionnaire (*n* = 159); Figure S1: Statistical output of all analyses. Appendix: The Stata output of all analyses.

## References

[1] France M.M., Turner J.R. (2017). The mucosal barrier at a glance. J. Cell Sci..

[2] Vancamelbeke M., Vermeire S. (2017). The intestinal barrier: A fundamental role in health and disease. Expert Rev. Gastroenterol. Hepatol..

[3] Schroeder B.O. (2019). Fight them or feed them: How the intestinal mucus layer manages the gut microbiota. Gastroenterol. Rep..

[4] Turner J.R. (2009). Intestinal mucosal barrier function in health and disease. Nat. Rev. Immunol..

[5] Pelaseyed T., Bergström J.H., Gustafsson J.K., Ermund A., Birchenough G.M.H., Schütte A., Post S., Svensson F., Rodríguez-Piñeiro A.M., Nyström E.E.L. (2014). The mucus and mucins of the goblet cells and enterocytes provide the first defense line of the gastrointestinal tract and interact with the immune system. Immunol. Rev..

[6] Allen A., Hutton D.A., Pearson J.P. (1998). The MUC2 gene product: A human intestinal mucin. Int. J. Biochem. Cell Biol..

[7] Johansson M.E., Hansson G.C. (2013). Mucus and the goblet cell. Dig. Dis..

[8] Wlodarska M., Thaiss C.A., Nowarski R., Henao-Mejia J., Zhang J.P., Brown E.M., Frankel G., Levy M., Katz M.N., Philbrick W.M. (2014). NLRP6 inflammasome orchestrates the colonic host-microbial interface by regulating goblet cell mucus secretion. Cell.

[9] Kim Y.S., Ho S.B. (2010). Intestinal goblet cells and mucins in health and disease: Recent insights and progress. Curr. Gastroenterol. Rep..

[10] Johansson M.E., Phillipson M., Petersson J., Velcich A., Holm L., Hansson G.C. (2008). The inner of the two Muc2 mucin-dependent mucus layers in colon is devoid of bacteria. Proc. Natl. Acad. Sci. USA.

[11] Johansson M.E., Sjovall H., Hansson G.C. (2013). The gastrointestinal mucus system in health and disease. Nat. Rev. Gastroenterol. Hepatol..

[12] Arnold J.W., Klimpel G.R., Niesel D.W. (1993). Tumor necrosis factor (TNF alpha) regulates intestinal mucus production during salmonellosis. Cell. Immunol..

[13] Merga Y., Campbell B.J., Rhodes J.M. (2014). Mucosal barrier, bacteria and inflammatory bowel disease: Possibilities for therapy. Dig. Dis..

[14] Van der Sluis M., De Koning B.A.E., De Bruijn A.C.J.M., Velcich A., Meijerink J.P.P., Van Goudoever J.B., Büller H.A., Dekker J., Van Seuningen I., Renes I.B. (2006). Muc2-Deficient Mice Spontaneously Develop Colitis, Indicating That MUC2 Is Critical for Colonic Protection. Gastroenterology.

[15] Chen S.J., Liu X.W., Liu J.P., Yang X.Y., Lu F.G. (2014). Ulcerative colitis as a polymicrobial infection characterized by sustained broken mucus barrier. World J. Gastroenterol..

[16] van der Post S., Jabbar K.S., Birchenough G., Arike L., Akhtar N., Sjovall H., Johansson M.E.V., Hansson G.C. (2019). Structural weakening of the colonic mucus barrier is an early event in ulcerative colitis pathogenesis. Gut.

[17] Gouyer V., Dubuquoy L., Robbe-Masselot C., Neut C., Singer E., Plet S., Geboes K., Desreumaux P., Gottrand F., Desseyn J.L. (2015). Delivery of a mucin domain enriched in cysteine residues strengthens the intestinal mucous barrier. Sci. Rep..

[18] Desseyn J.L., Gouyer V., Gottrand F. (2016). Biological modeling of mucus to modulate mucus barriers. Am. J. Physiol. Gastrointest. Liver Physiol..

[19] Sun J., Shen X., Li Y., Guo Z., Zhu W., Zuo L., Zhao J., Gu L., Gong J., Li J. (2016). Therapeutic Potential to Modify the Mucus Barrier in Inflammatory Bowel Disease. Nutrients.

[20] Andersen V., Holmskov U., Sørensen S.B., Jawhara M., Andersen K.W., Bygum A., Hvid L., Grauslund J., Wied J., Glerup H. (2017). A Proposal for a Study on Treatment Selection and Lifestyle Recommendations in Chronic Inflammatory Diseases: A Danish Multidisciplinary Collaboration on Prognostic Factors and Personalised Medicine. Nutrients.

[21] Christensen R., Heitmann B.L., Andersen K.W., Nielsen O.H., Sorensen S.B., Jawhara M., Bygum A., Hvid L., Grauslund J., Wied J. (2018). Impact of red and processed meat and fibre intake on treatment outcomes among patients with chronic inflammatory diseases: Protocol for a prospective cohort study of prognostic factors and personalised medicine. BMJ Open.

[22] Jowett S.L., Seal C.J., Pearce M.S., Phillips E., Gregory W., Barton J.R., Welfare M.R. (2004). Influence of dietary factors on the clinical course of ulcerative colitis: A prospective cohort study. Gut.

[23] Pattison D.J., Symmons D.P., Lunt M., Welch A., Luben R., Bingham S.A., Khaw K.T., Day N.E., Silman A.J. (2004). Dietary risk factors for the development of inflammatory polyarthritis: Evidence for a role of high level of red meat consumption. Arthritis Rheum..

[24] Magee E.A., Richardson C.J., Hughes R., Cummings J.H. (2000). Contribution of dietary protein to sulfide production in the large intestine: An in vitro and a controlled feeding study in humans. Am. J. Clin. Nutr..

[25] Kushkevych I., Dordevic D., Vitezova M. (2019). Toxicity of hydrogen sulfide toward sulfate-reducing bacteria Desulfovibrio piger Vib-7. Arch. Microbiol..

[26] Ijssennagger N., van der Meer R., van Mil S.W. (2016). Sulfide as a Mucus Barrier-Breaker in Inflammatory Bowel Disease?. Trends Mol. Med..

[27] Bastide N.M., Chenni F., Audebert M., Santarelli R.L., Tache S., Naud N., Baradat M., Jouanin I., Surya R., Hobbs D.A. (2015). A central role for heme iron in colon carcinogenesis associated with red meat intake. Cancer Res..

[28] Gamage S.M.K., Dissabandara L., Lam A.K., Gopalan V. (2018). The role of heme iron molecules derived from red and processed meat in the pathogenesis of colorectal carcinoma. Crit. Rev. Oncol. Hematol..

[29] Minihane A.M., Vinoy S., Russell W.R., Baka A., Roche H.M., Tuohy K.M., Teeling J.L., Blaak E.E., Fenech M., Vauzour D. (2015). Low-grade inflammation, diet composition and health: Current research evidence and its translation. Br. J. Nutr..

[30] Seal C.J., Brownlee I.A. (2015). Whole-grain foods and chronic disease: Evidence from epidemiological and intervention studies. Proc. Nutr. Soc..

[31] Roager H.M., Vogt J.K., Kristensen M., Hansen L.B.S., Ibrugger S., Maerkedahl R.B., Bahl M.I., Lind M.V., Nielsen R.L., Frokiaer H. (2019). Whole grain-rich diet reduces body weight and systemic low-grade inflammation without inducing major changes of the gut microbiome: A randomised cross-over trial. Gut.

[32] Suzuki T., Yoshida S., Hara H. (2008). Physiological concentrations of short-chain fatty acids immediately suppress colonic epithelial permeability. Br. J. Nutr..

[33] van der Beek C.M., Dejong C.H.C., Troost F.J., Masclee A.A.M., Lenaerts K. (2017). Role of short-chain fatty acids in colonic inflammation, carcinogenesis, and mucosal protection and healing. Nutr. Rev..

[34] Wong J.M., de Souza R., Kendall C.W., Emam A., Jenkins D.J. (2006). Colonic health: Fermentation and short chain fatty acids. J. Clin. Gastroenterol..

[35] Jawhara M.S.S.B. (2018). Hvem har Gavn af Medicinsk Behandling Med Præparater Rettet Mod TNF-α. Gastroenterol. Best Pract. Nord..

[36] e-Boks. https://www.nets.eu/developer/Nets%20share/ebokschannel/Pages/default.aspx.

[37] Lewis S.J., Heaton K.W. (1997). Stool form scale as a useful guide to intestinal transit time. Scand. J. Gastroenterol..

[38] Ware J., Kosinski M., Keller S.D. (1996). A 12-Item Short-Form Health Survey: Construction of scales and preliminary tests of reliability and validity. Med Care.

[39] Eriksen L., Gronbaek M., Helge J.W., Tolstrup J.S., Curtis T. (2011). The Danish Health Examination Survey 2007-2008 (DANHES 2007-2008). Scand. J. Public Health.

[40] National Food Institute, Technical University of Denmark Danish Food Composition Tables. https://frida.fooddata.dk/?lang=en.

[41] Harris P.A., Taylor R., Thielke R., Payne J., Gonzalez N., Conde J.G. (2009). Research electronic data capture (REDCap)—A metadata-driven methodology and workflow process for providing translational research informatics support. J. Biomed. Inform..

[42] Harris P.A., Taylor R., Minor B.L., Elliott V., Fernandez M., O’Neal L., McLeod L., Delacqua G., Delacqua F., Kirby J. (2019). The REDCap consortium: Building an international community of software platform partners. J. Biomed. Inform..

[43] Network, O.P.d.E. https://www.sdu.dk/en/om_sdu/institutter_centre/klinisk_institut/forskning/forskningsenheder/open.Aspx.

[44] Tenekedjiev K.I., Nikolova N.D., Kolev K., Mode C.J. (2011). Wellcome Trust-Funded Monographs and Book Chapters Applications of Monte Carlo Simulation in Modelling of Biochemical Processes. Applications of Monte Carlo Methods in Biology, Medicine and Other Fields of Science.

[45] StataCorp (2015). Stata Statistical Software: Release 15.

[46] Cohen M., Varki N.M., Jankowski M.D., Gagneux P. (2012). Using Unfixed, Frozen Tissues to Study Natural Mucin Distribution. J. Vis. Exp. Jove.

[47] Johansson M.E.V., Hansson G.C., McGuckin M.A., Thornton D.J. (2012). Preservation of Mucus in Histological Sections, Immunostaining of Mucins in Fixed Tissue, and Localization of Bacteria with FISH. Mucins: Methods and Protocols.

[48] Amann R.I., Binder B.J., Olson R.J., Chisholm S.W., Devereux R., Stahl D.A. (1990). Combination of 16S rRNA-targeted oligonucleotide probes with flow cytometry for analyzing mixed microbial populations. Appl. Environ. Microbiol..

[49] Jensen H.E., Jensen L.K., Barington K., Pors S.E., Bjarnsholt T., Boye M. (2015). Fluorescence in situ hybridization for the tissue detection of bacterial pathogens associated with porcine infections. Methods Mol. Biol. (Clifton N. J.).

[50] Hamamatsu.com: NDP.view2 U12388-01. https://www.hamamatsu.com/eu/en/product/type/U12388-01/index.html.

[51] Bruun N.H. (2015). SF12: Stata Module to Validate sf12 Input and Calculate sf12 Version 2 t Scores. https://ideas.repec.org/c/boc/bocode/s458125.html.

[52] O’Donnell L.J., Virjee J., Heaton K.W. (1990). Detection of pseudodiarrhoea by simple clinical assessment of intestinal transit rate. BMJ (Clin. Res. Ed.).

[53] Chan D.S., Lau R., Aune D., Vieira R., Greenwood D.C., Kampman E., Norat T. (2011). Red and processed meat and colorectal cancer incidence: Meta-analysis of prospective studies. PLoS ONE.

[54] Sasso A., Latella G. (2018). Role of Heme Iron in the Association Between Red Meat Consumption and Colorectal Cancer. Nutr. Cancer.

[55] Gibson G.R., Macfarlane G.T., Cummings J.H. (1993). Sulphate reducing bacteria and hydrogen metabolism in the human large intestine. Gut.

[56] Saunders B.P., Fukumoto M., Halligan S., Jobling C., Moussa M.E., Bartram C.I., Williams C.B. (1996). Why is colonoscopy more difficult in women?. Gastrointest. Endosc..

[57] Phillips M., Patel A., Meredith P., Will O., Brassett C. (2015). Segmental colonic length and mobility. Ann. R. Coll. Surg. Engl..

[58] Elderman M., Sovran B., Hugenholtz F., Graversen K., Huijskes M., Houtsma E., Belzer C., Boekschoten M., de Vos P., Dekker J. (2017). The effect of age on the intestinal mucus thickness, microbiota composition and immunity in relation to sex in mice. PLoS ONE.

[59] Cummings J.H., Hill M.J., Bone E.S., Branch W.J., Jenkins D.J. (1979). The effect of meat protein and dietary fiber on colonic function and metabolism. II. Bacterial metabolites in feces and urine. Am. J. Clin. Nutr..

[60] Meldrum O.W., Yakubov G.E., Gartaula G., McGuckin M.A., Gidley M.J. (2017). Mucoadhesive functionality of cell wall structures from fruits and grains: Electrostatic and polymer network interactions mediated by soluble dietary polysaccharides. Sci. Rep..

[61] Bucher P., Gervaz P., Egger J.F., Soravia C., Morel P. (2006). Morphologic alterations associated with mechanical bowel preparation before elective colorectal surgery: A randomized trial. Dis. Colon Rectum.

[62] Coskun A., Uzunkoy A., Duzgun S.A., Bozer M., Ozardali I., Vural H. (2001). Experimental sodium phosphate and polyethylene glycol induce colonic tissue damage and oxidative stress. BJS (Br. J. Surg.).

[63] Atuma C., Strugala V., Allen A., Holm L. (2001). The adherent gastrointestinal mucus gel layer: Thickness and physical state in vivo. Am. J. Physiol. Gastrointest. Liver Physiol..

[64] Johansson M.E., Larsson J.M., Hansson G.C. (2011). The two mucus layers of colon are organized by the MUC2 mucin, whereas the outer layer is a legislator of host-microbial interactions. Proc. Natl. Acad. Sci. USA.

[65] Hasegawa Y., Mark Welch J.L., Rossetti B.J., Borisy G.G. (2017). Preservation of three-dimensional spatial structure in the gut microbiome. PLoS ONE.

[66] Zhu J., Ma S., Xiao P., Li L., Yang Y. (2015). Meta-analysis on the relationship among fiber of grain and intestinal motility and symptoms. Wei Sheng Yan Jiu J. Hyg. Res..

[67] James L.R., Brett J.M. (1984). Mediators, moderators, and tests for mediation. J. Appl. Psychol..

[68] MacKinnon D.P., Krull J.L., Lockwood C.M. (2000). Equivalence of the mediation, confounding and suppression effect. Prev. Sci..

[69] Paturi G., Butts C.A., Bentley-Hewitt K.L., Hedderley D., Stoklosinski H., Ansell J. (2015). Differential effects of probiotics, prebiotics, and synbiotics on gut microbiota and gene expression in rats. J. Funct. Foods.

[70] Kyrø C., Skeie G., Dragsted L.O., Christensen J., Overvad K., Hallmans G., Johansson I., Lund E., Slimani N., Johnsen N.F. (2012). Intake of whole grain in Scandinavia: Intake, sources and compliance with new national recommendations. Scand. J. Public Health.

[71] Jawhara M., Sørensen S.B., Heitmann B.L., Andersen V. (2019). Biomarkers of Whole-Grain and Cereal-Fiber Intake in Human Studies: A Systematic Review of the Available Evidence and Perspectives. Nutrients.

[72] Cuparencu C., Praticó G., Hemeryck L.Y., Sri Harsha P.S.C., Noerman S., Rombouts C., Xi M., Vanhaecke L., Hanhineva K., Brennan L. (2019). Biomarkers of meat and seafood intake: An extensive literature review. Genes Nutr..

