# Supplementary material for: The Relation between Red Meat and Whole-Grain Intake and the Colonic Mucosal Barrier: A Cross-Sectional Study
Source: Nutrients. 2020 Jun 12;12(6):1765. doi: 10.3390/nu12061765 (PMC7353246; doi:10.3390/nu12061765)
Supplement: Supplementary file 1 [file nutrients-12-01765-s001.zip › Supplementary materials/Figure S1.pdf]

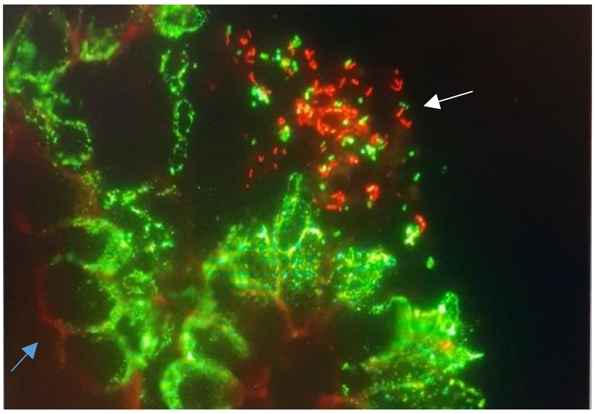

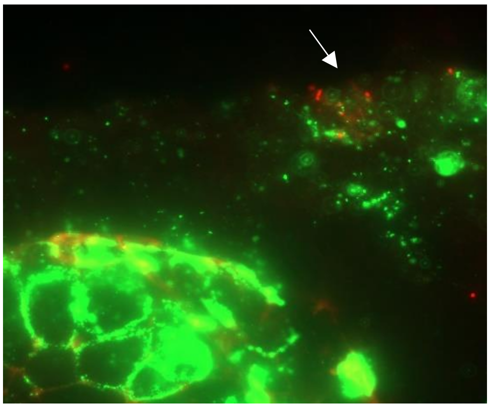

(**a**) (**b**)

Figure S1. These images represent the immunostaining and fluorescence in situ hybridization stained mucus of the colon sigmoideum from two different subjects. The bacterial strains are stained in red and indicated by the white arrows in (a) and (b). Mucus is stained with green in (a) and (b). A diffuse cross-reaction was observed in the area indicated by the blue arrow in (a).
