# Supplementary material for: The Relation between Red Meat and Whole-Grain Intake and the Colonic Mucosal Barrier: A Cross-Sectional Study
Source: Nutrients. 2020 Jun 12;12(6):1765. doi: 10.3390/nu12061765 (PMC7353246; doi:10.3390/nu12061765)
Supplement: Supplementary file 1 [file nutrients-12-01765-s001.zip › Supplementary materials/Stata output 1.pdf]

```
. do "C:\Users\pek3at\AppData\Local\Temp\STD1a44_000000.tmp"

. replace Whole_grains = . if energi<1600 | energi>30000
(3 real changes made, 3 to missing)

. replace RedMeat  = . if energi<1600 | energi>30000
(3 real changes made, 3 to missing)

. mi set mlong

. mi register imputed RedMeat Whole_grains
(3 m=0 obs. now marked as incomplete)

. mi impute mvn RedMeat Whole_grains = fats kfibre age sex_intro, add(100) rseed(20200113)

Performing EM optimization:
note: 3 observations omitted from EM estimation because of all imputation variables missing
  observed log likelihood = -1350.3733 at iteration 1

Performing MCMC data augmentation ...

Multivariate imputation                     Imputations =       100
Multivariate normal regression                    added =       100
Imputed: m=1 through m=100                      updated =         0

Prior: uniform                               Iterations =     10000
                                                burn-in =       100
                                                between =       100

------------------------------------------------------------------
                   |               Observations per m
                   |----------------------------------------------
          Variable |   Complete   Incomplete   Imputed |     Total
-------------------+-----------------------------------+----------
           RedMeat |        156            3         3 |       159
      Whole_grains |        156            3         3 |       159
------------------------------------------------------------------
(complete + incomplete = total; imputed is the minimum across m
 of the number of filled-in observations.)

. mi reshape long tyk, i(patient_id) j(measurement)

reshaping m=0 data ...
(note: j = 1 2 3 4 5)

Data                               wide   ->   long
-----------------------------------------------------------------------------
Number of obs.                      159   ->     795
Number of variables                 133   ->     130
j variable (5 values)                     ->   measurement
xij variables:
                     tyk1 tyk2 ... tyk5   ->   tyk
-----------------------------------------------------------------------------

reshaping m=1 data ...

reshaping m=2 data ...

reshaping m=3 data ...

reshaping m=4 data ...

reshaping m=5 data ...

reshaping m=6 data ...

reshaping m=7 data ...

reshaping m=8 data ...

reshaping m=9 data ...
```

reshaping *m*=10 data ...

reshaping *m*=11 data ...

reshaping *m*=12 data ...

reshaping *m*=13 data ...

reshaping *m*=14 data ...

reshaping *m*=15 data ...

reshaping *m*=16 data ...

reshaping *m*=17 data ...

reshaping *m*=18 data ...

reshaping *m*=19 data ...

reshaping *m*=20 data ...

reshaping *m*=21 data ...

reshaping *m*=22 data ...

reshaping *m*=23 data ...

reshaping *m*=24 data ...

reshaping *m*=25 data ...

reshaping *m*=26 data ...

reshaping *m*=27 data ...

reshaping *m*=28 data ...

reshaping *m*=29 data ...

reshaping *m*=30 data ...

reshaping *m*=31 data ...

reshaping *m*=32 data ...

reshaping *m*=33 data ...

reshaping *m*=34 data ...

reshaping *m*=35 data ...

reshaping *m*=36 data ...

reshaping *m*=37 data ...

reshaping *m*=38 data ...

reshaping *m*=39 data ...

reshaping *m*=40 data ...

reshaping *m*=41 data ...

reshaping *m*=42 data ...

reshaping *m*=43 data ...

reshaping *m*=44 data ...

reshaping *m*=45 data ...

reshaping *m*=46 data ...

reshaping *m*=47 data ...

reshaping *m*=48 data ...

reshaping *m*=49 data ...

reshaping *m*=50 data ...

reshaping *m*=51 data ...

reshaping *m*=52 data ...

reshaping *m*=53 data ...

reshaping *m*=54 data ...

reshaping *m*=55 data ...

reshaping *m*=56 data ...

reshaping *m*=57 data ...

reshaping *m*=58 data ...

reshaping *m*=59 data ...

reshaping *m*=60 data ...

reshaping *m*=61 data ...

reshaping *m*=62 data ...

reshaping *m*=63 data ...

reshaping *m*=64 data ...

reshaping *m*=65 data ...

reshaping *m*=66 data ...

reshaping *m*=67 data ...

reshaping *m*=68 data ...

reshaping *m*=69 data ...

reshaping *m*=70 data ...

reshaping *m*=71 data ...

reshaping *m*=72 data ...

reshaping *m*=73 data ...

reshaping *m*=74 data ...

reshaping *m*=75 data ...

reshaping *m*=76 data ...

reshaping *m*=77 data ...

reshaping *m*=78 data ...

reshaping *m*=79 data ...

reshaping *m*=80 data ...

reshaping *m*=81 data ...

reshaping *m*=82 data ...

reshaping *m*=83 data ...

```
reshaping m=84 data ...

reshaping m=85 data ...

reshaping m=86 data ...

reshaping m=87 data ...

reshaping m=88 data ...

reshaping m=89 data ...

reshaping m=90 data ...

reshaping m=91 data ...

reshaping m=92 data ...

reshaping m=93 data ...

reshaping m=94 data ...

reshaping m=95 data ...

reshaping m=96 data ...

reshaping m=97 data ...

reshaping m=98 data ...

reshaping m=99 data ...

reshaping m=100 data ...

assembling results ...

. gen logtyk=log(tyk)
(1,064 missing values generated)

. mi estimate: mixed logtyk Whole_grains ||patient_id:

Multiple-imputation estimates                Imputations       =        100
Mixed-effects ML regression                  Number of obs     =        131

Group variable: patient_id                   Number of groups  =         39
                                             Obs per group:
                                                           min =          3
                                                           avg =        3.4
                                                           max =          5
                                             Average RVI       =     0.0025
                                             Largest FMI       =     0.0045
DF adjustment:   Large sample                DF:     min       = 4979059.62
                                                     avg       =   2.94e+18
                                                     max       =   1.18e+19
Model F test:       Equal FMI                F(   1, 5.0e+06)  =       9.24
                                             Prob > F          =     0.0024
```

logtyk	Coef.	Std. Err.	t	P>|t|	[95% Conf.	Interval]
Whole_grains	-.0012262	.0004034	-3.04	0.002	-.0020169	-.0004355
_cons	3.084366	.1032196	29.88	0.000	2.882059	3.286673

Random-effects Parameters	Estimate	Std. Err.	[95% Conf.	Interval]
**patient_id**: Identity				
sd(_cons)	.4366279	.05003	.3488016	.5465683
sd(Residual)	.0805347	.0059362	.0697013	.0930518

```
. mi estimate: mixed logtyk RedMeat ||patient_id:

Multiple-imputation estimates                   Imputations       =        100
Mixed-effects ML regression                     Number of obs     =        131

Group variable: patient_id                      Number of groups  =         39
                                                Obs per group:
                                                              min =          3
                                                              avg =        3.4
                                                              max =          5
                                                Average RVI       =     0.0092
                                                Largest FMI       =     0.0263
DF adjustment:   Large sample                   DF:     min       = 143,814.11
                                                        avg       =   7.29e+17
                                                        max       =   2.91e+18
Model F test:       Equal FMI                   F(   1,143814.1)  =       5.17
                                                Prob > F          =     0.0229
```

logtyk	Coef.	Std. Err.	t	P>|t|	[95% Conf.	Interval]
RedMeat	-.0018136	.0007974	-2.27	0.023	-.0033764	-.0002508
_cons	3.070236	.119624	25.67	0.000	2.835777	3.304695

Random-effects Parameters	Estimate	Std. Err.	[95% Conf.	Interval]
**patient_id**: Identity				
sd(_cons)	.4557952	.0522634	.3640552	.5706532
sd(Residual)	.0805324	.0059357	.0696999	.0930485

```
. mi estimate: mixed logtyk RedMeat Whole_grains ||patient_id:

Multiple-imputation estimates                   Imputations       =        100
Mixed-effects ML regression                     Number of obs     =        131

Group variable: patient_id                      Number of groups  =         39
                                                Obs per group:
                                                              min =          3
                                                              avg =        3.4
                                                              max =          5
                                                Average RVI       =     0.0117
                                                Largest FMI       =     0.0358
DF adjustment:   Large sample                   DF:     min       =  77,395.46
                                                        avg       =   2.81e+17
                                                        max       =   1.40e+18
Model F test:       Equal FMI                   F(   2,327004.5)  =       6.66
                                                Prob > F          =     0.0013
```

logtyk	Coef.	Std. Err.	t	P>|t|	[95% Conf.	Interval]
RedMeat	-.0013894	.0007523	-1.85	0.065	-.0028638	.0000851
Whole_grains	-.001068	.0003967	-2.69	0.007	-.0018455	-.0002904
_cons	3.219972	.1233151	26.11	0.000	2.978278	3.461665

Random-effects Parameters	Estimate	Std. Err.	[95% Conf.	Interval]
**patient_id**: Identity				
sd(_cons)	.4177099	.0479624	.3335323	.5231325
sd(Residual)	.0805336	.005936	.0697006	.0930502

```
. mi estimate: mixed logtyk RedMeat Whole_grains age ||patient_id:

Multiple-imputation estimates                   Imputations       =        100
Mixed-effects ML regression                     Number of obs     =        131

Group variable: patient_id                      Number of groups  =         39
                                                Obs per group:
                                                              min =          3
                                                              avg =        3.4
                                                              max =          5
                                                Average RVI       =     0.0128
                                                Largest FMI       =     0.0405
DF adjustment:   Large sample                   DF:     min       =  60,464.39
                                                        avg       =   2.14e+17
                                                        max       =   1.29e+18
Model F test:       Equal FMI                   F(   3,694967.3)  =       5.87
                                                Prob > F          =     0.0005

------------------------------------------------------------------------------
      logtyk |      Coef.   Std. Err.      t    P>|t|     [95% Conf. Interval]
-------------+----------------------------------------------------------------
     RedMeat |  -.0017142   .0007482    -2.29   0.022    -.0031807   -.0002478
Whole_grains |  -.0010989   .0003822    -2.88   0.004     -.001848   -.0003498
         age |   .0114757   .0064213     1.79   0.074    -.0011099    .0240612
       _cons |   2.518586   .4097513     6.15   0.000     1.715488    3.321684
------------------------------------------------------------------------------

------------------------------------------------------------------------------
  Random-effects Parameters  |   Estimate   Std. Err.     [95% Conf. Interval]
-----------------------------+------------------------------------------------
patient_id: Identity         |
                   sd(_cons) |   .4012759   .0462076      .3202032    .5028755
-----------------------------+------------------------------------------------
                sd(Residual) |   .0805353   .0059364      .0697017    .0930528
------------------------------------------------------------------------------

. mi estimate: mixed logtyk RedMeat Whole_grains age sex_intro ||patient_id:

Multiple-imputation estimates                   Imputations       =        100
Mixed-effects ML regression                     Number of obs     =        131

Group variable: patient_id                      Number of groups  =         39
                                                Obs per group:
                                                              min =          3
                                                              avg =        3.4
                                                              max =          5
                                                Average RVI       =     0.0117
                                                Largest FMI       =     0.0414
DF adjustment:   Large sample                   DF:     min       =  57,902.16
                                                        avg       =   2.71e+17
                                                        max       =   1.89e+18
Model F test:       Equal FMI                   F(   4, 1.4e+06)  =       4.53
                                                Prob > F          =     0.0012

------------------------------------------------------------------------------
      logtyk |      Coef.   Std. Err.      t    P>|t|     [95% Conf. Interval]
-------------+----------------------------------------------------------------
     RedMeat |  -.0016229   .0007661    -2.12   0.034    -.0031244   -.0001213
Whole_grains |  -.0010801   .0003824    -2.82   0.005    -.0018295   -.0003307
         age |   .0128912   .0069179     1.86   0.062    -.0006676      .02645
   sex_intro |     .08163   .1509543     0.54   0.589     -.214235     .377495
       _cons |    2.29705   .5791206     3.97   0.000     1.161994    3.432107
------------------------------------------------------------------------------

------------------------------------------------------------------------------
  Random-effects Parameters  |   Estimate   Std. Err.     [95% Conf. Interval]
-----------------------------+------------------------------------------------
patient_id: Identity         |
                   sd(_cons) |   .3997167    .046037       .318945    .5009436
-----------------------------+------------------------------------------------
                sd(Residual) |   .0805363   .0059366      .0697023    .0930543
------------------------------------------------------------------------------
```

```
. mi estimate: mixed logtyk RedMeat Whole_grains bmi ||patient_id:

Multiple-imputation estimates                   Imputations       =        100
Mixed-effects ML regression                     Number of obs     =        121

Group variable: patient_id                      Number of groups  =         36
                                                Obs per group:
                                                              min =          3
                                                              avg =        3.4
                                                              max =          5
                                                Average RVI       =     0.0011
                                                Largest FMI       =     0.0031
DF adjustment:   Large sample                   DF:     min       =   1.01e+07
                                                        avg       =   2.57e+20
                                                        max       =   1.54e+21
Model F test:       Equal FMI                   F(   3, 9.3e+07)  =       5.16
                                                Prob > F          =     0.0014

------------------------------------------------------------------------------
      logtyk |      Coef.   Std. Err.      t    P>|t|     [95% Conf. Interval]
-------------+----------------------------------------------------------------
     RedMeat |  -.0011617   .0007907    -1.47   0.142    -.0027115     .000388
Whole_grains |   -.001144   .0003952    -2.89   0.004    -.0019186   -.0003694
         bmi |  -.0453095   .0227532    -1.99   0.046    -.0899048   -.0007141
       _cons |   4.439307   .6343258     7.00   0.000     3.196052    5.682563
------------------------------------------------------------------------------

------------------------------------------------------------------------------
  Random-effects Parameters  |   Estimate   Std. Err.     [95% Conf. Interval]
-----------------------------+------------------------------------------------
patient_id: Identity         |
                   sd(_cons) |   .3958148   .0472401      .3132579    .5001289
-----------------------------+------------------------------------------------
                sd(Residual) |   .0808353   .0061992      .0695542    .0939462
------------------------------------------------------------------------------

. mi estimate: mixed logtyk RedMeat Whole_grains age sex_intro bmi ||patient_id:

Multiple-imputation estimates                   Imputations       =        100
Mixed-effects ML regression                     Number of obs     =        121

Group variable: patient_id                      Number of groups  =         36
                                                Obs per group:
                                                              min =          3
                                                              avg =        3.4
                                                              max =          5
                                                Average RVI       =     0.0013
                                                Largest FMI       =     0.0024
DF adjustment:   Large sample                   DF:     min       =   1.75e+07
                                                        avg       =   9.13e+17
                                                        max       =   7.30e+18
Model F test:       Equal FMI                   F(   5, 1.5e+08)  =       5.89
                                                Prob > F          =     0.0000

------------------------------------------------------------------------------
      logtyk |      Coef.   Std. Err.      t    P>|t|     [95% Conf. Interval]
-------------+----------------------------------------------------------------
     RedMeat |  -.0010367   .0007356    -1.41   0.159    -.0024786    .0004051
Whole_grains |  -.0009924   .0003555    -2.79   0.005    -.0016892   -.0002956
         age |   .0245749   .0079127     3.11   0.002     .0090663    .0400836
   sex_intro |    .222672   .1394869     1.60   0.110    -.0507173    .4960613
         bmi |  -.0463002   .0202309    -2.29   0.022    -.0859521   -.0066484
       _cons |   2.488979    .846712     2.94   0.003     .8294537    4.148504
------------------------------------------------------------------------------

------------------------------------------------------------------------------
  Random-effects Parameters  |   Estimate   Std. Err.     [95% Conf. Interval]
-----------------------------+------------------------------------------------
patient_id: Identity         |
                   sd(_cons) |   .3504039   .0419703      .2770861    .4431218
-----------------------------+------------------------------------------------
                sd(Residual) |   .0808395   .0062001      .0695567    .0939525
------------------------------------------------------------------------------
```

```
. mi estimate: mixed logtyk RedMeat Whole_grains bmi ||patient_id:

Multiple-imputation estimates                   Imputations       =        100
Mixed-effects ML regression                     Number of obs     =        121

Group variable: patient_id                      Number of groups  =         36
                                                Obs per group:
                                                              min =          3
                                                              avg =        3.4
                                                              max =          5
                                                Average RVI       =     0.0011
                                                Largest FMI       =     0.0031
DF adjustment:   Large sample                   DF:     min       =   1.01e+07
                                                        avg       =   2.57e+20
                                                        max       =   1.54e+21
Model F test:       Equal FMI                   F(   3, 9.3e+07)  =       5.16
                                                Prob > F          =     0.0014

------------------------------------------------------------------------------
      logtyk |      Coef.   Std. Err.      t    P>|t|     [95% Conf. Interval]
-------------+----------------------------------------------------------------
     RedMeat |  -.0011617   .0007907    -1.47   0.142    -.0027115     .000388
Whole_grains |   -.001144   .0003952    -2.89   0.004    -.0019186   -.0003694
         bmi |  -.0453095   .0227532    -1.99   0.046    -.0899048   -.0007141
       _cons |   4.439307   .6343258     7.00   0.000     3.196052    5.682563
------------------------------------------------------------------------------

------------------------------------------------------------------------------
  Random-effects Parameters  |   Estimate   Std. Err.     [95% Conf. Interval]
-----------------------------+------------------------------------------------
patient_id: Identity         |
                   sd(_cons) |   .3958148   .0472401      .3132579    .5001289
-----------------------------+------------------------------------------------
                sd(Residual) |   .0808353   .0061992      .0695542    .0939462
------------------------------------------------------------------------------

.
end of do-file

.
```
