# Supplementary material for: The Relation between Red Meat and Whole-Grain Intake and the Colonic Mucosal Barrier: A Cross-Sectional Study
Source: Nutrients. 2020 Jun 12;12(6):1765. doi: 10.3390/nu12061765 (PMC7353246; doi:10.3390/nu12061765)
Supplement: Supplementary file 1 [file nutrients-12-01765-s001.zip › Supplementary materials/Stata output2.pdf]

```
. do "C:\Users\pek3at\AppData\Local\Temp\STD1a44_000000.tmp"

. replace Whole_grains = . if energi<1600 | energi>30000
(3 real changes made, 3 to missing)

. replace RedMeat  = . if energi<1600 | energi>30000
(3 real changes made, 3 to missing)

. reshape long tyk, i(kost) j(measurement)
(note: j = 1 2 3 4 5)

Data                               wide   ->   long
-----------------------------------------------------------------------------
Number of obs.                      159   ->     795
Number of variables                 133   ->     130
j variable (5 values)                     ->   measurement
xij variables:
                     tyk1 tyk2 ... tyk5   ->   tyk
-----------------------------------------------------------------------------

. gen logtyk=log(tyk)
(664 missing values generated)

. mixed logtyk RedMeat Whole_grains ||patient_id:

Performing EM optimization:

Performing gradient-based optimization:

Iteration 0:   log likelihood =  49.676225
Iteration 1:   log likelihood =  49.676225

Computing standard errors:

Mixed-effects ML regression                     Number of obs     =        120
Group variable: patient_id                      Number of groups  =         36

                                                Obs per group:
                                                              min =          3
                                                              avg =        3.3
                                                              max =          5

                                                Wald chi2(2)      =       8.91
Log likelihood =  49.676225                     Prob > chi2       =     0.0116

------------------------------------------------------------------------------
      logtyk |      Coef.   Std. Err.      z    P>|z|     [95% Conf. Interval]
-------------+----------------------------------------------------------------
     RedMeat |  -.0013657   .0008031    -1.70   0.089    -.0029398    .0002084
Whole_grains |  -.0009735   .0004147    -2.35   0.019    -.0017862   -.0001608
       _cons |    3.16485   .1364667    23.19   0.000      2.89738     3.43232
------------------------------------------------------------------------------

------------------------------------------------------------------------------
  Random-effects Parameters  |   Estimate   Std. Err.     [95% Conf. Interval]
-----------------------------+------------------------------------------------
patient_id: Identity         |
                  var(_cons) |   .1691862   .0403444      .1060195    .2699877
-----------------------------+------------------------------------------------
               var(Residual) |   .0067933    .001048      .0050208    .0091916
------------------------------------------------------------------------------
LR test vs. linear model: chibar2(01) = 237.42        Prob >= chibar2 = 0.0000
```

```
. mixed logtyk RedMeat Whole_grains age||patient_id:

Performing EM optimization:

Performing gradient-based optimization:

Iteration 0:   log likelihood =  51.249972
Iteration 1:   log likelihood =  51.249972

Computing standard errors:

Mixed-effects ML regression                     Number of obs     =        120
Group variable: patient_id                      Number of groups  =         36

                                                Obs per group:
                                                              min =          3
                                                              avg =        3.3
                                                              max =          5

                                                Wald chi2(3)      =      13.02
Log likelihood =  51.249972                     Prob > chi2       =     0.0046

------------------------------------------------------------------------------
      logtyk |      Coef.   Std. Err.      z    P>|z|     [95% Conf. Interval]
-------------+----------------------------------------------------------------
     RedMeat |  -.0016605   .0007857    -2.11   0.035    -.0032005   -.0001205
Whole_grains |  -.0010012   .0003972    -2.52   0.012    -.0017797   -.0002227
         age |   .0117288   .0064667     1.81   0.070    -.0009458    .0244033
       _cons |   2.446765    .416918     5.87   0.000     1.629621     3.26391
------------------------------------------------------------------------------

------------------------------------------------------------------------------
  Random-effects Parameters  |   Estimate   Std. Err.     [95% Conf. Interval]
-----------------------------+------------------------------------------------
patient_id: Identity         |
                  var(_cons) |   .1548402   .0369671      .0969761    .2472308
-----------------------------+------------------------------------------------
               var(Residual) |   .0067935    .001048      .0050209    .0091919
------------------------------------------------------------------------------
LR test vs. linear model: chibar2(01) = 229.17        Prob >= chibar2 = 0.0000

. mixed logtyk RedMeat Whole_grains age sex_intro ||patient_id:

Performing EM optimization:

Performing gradient-based optimization:

Iteration 0:   log likelihood =  51.647745
Iteration 1:   log likelihood =  51.647745

Computing standard errors:

Mixed-effects ML regression                     Number of obs     =        120
Group variable: patient_id                      Number of groups  =         36

                                                Obs per group:
                                                              min =          3
                                                              avg =        3.3
                                                              max =          5

                                                Wald chi2(4)      =      14.11
Log likelihood =  51.647745                     Prob > chi2       =     0.0069

------------------------------------------------------------------------------
      logtyk |      Coef.   Std. Err.      z    P>|z|     [95% Conf. Interval]
-------------+----------------------------------------------------------------
     RedMeat |   -.001491   .0007997    -1.86   0.062    -.0030584    .0000764
Whole_grains |  -.0009686   .0003945    -2.46   0.014    -.0017418   -.0001954
         age |   .0146142   .0071589     2.04   0.041      .000583    .0286453
   sex_intro |   .1403961   .1565314     0.90   0.370    -.1663998     .447192
       _cons |   2.035765     .61642     3.30   0.001     .8276037    3.243926
------------------------------------------------------------------------------
```

Random-effects Parameters	Estimate	Std. Err.	[95% Conf.	Interval]
**patient_id**: Identity				
var(_cons)	**.1514004**	**.0361592**	**.0948054**	**.2417803**
var(Residual)	**.0067937**	**.0010481**	**.005021**	**.0091922**

LR test vs. linear model: chibar2(01) = **226.60**   Prob >= chibar2 = **0.0000**

```
. mixed logtyk RedMeat Whole_grains bmi ||patient_id:

Performing EM optimization:

Performing gradient-based optimization:

Iteration 0:   log likelihood =  50.209524
Iteration 1:   log likelihood =  50.209524

Computing standard errors:

Mixed-effects ML regression                     Number of obs     =        117
Group variable: patient_id                      Number of groups  =         35

                                                Obs per group:
                                                              min =          3
                                                              avg =        3.3
                                                              max =          5

                                                Wald chi2(3)      =      12.15
Log likelihood =  50.209524                     Prob > chi2       =     0.0069
```

logtyk	Coef.	Std. Err.	z	P>|z|	[95% Conf.	Interval]
RedMeat	**-.0011363**	**.000803**	**-1.41**	**0.157**	**-.0027101**	**.0004376**
Whole_grains	**-.0010952**	**.0004113**	**-2.66**	**0.008**	**-.0019013**	**-.000289**
bmi	**-.0419001**	**.0238749**	**-1.75**	**0.079**	**-.0886941**	**.0048939**
_cons	**4.326658**	**.6762727**	**6.40**	**0.000**	**3.001188**	**5.652128**

Random-effects Parameters	Estimate	Std. Err.	[95% Conf.	Interval]
**patient_id**: Identity				
var(_cons)	**.1598137**	**.0386807**	**.0994472**	**.256824**
var(Residual)	**.0066926**	**.0010451**	**.0049281**	**.0090889**

LR test vs. linear model: chibar2(01) = **225.23**   Prob >= chibar2 = **0.0000**

```
. mixed logtyk RedMeat Whole_grains age sex_intro bmi ||patient_id:

Performing EM optimization:

Performing gradient-based optimization:

Iteration 0:   log likelihood =  54.327539
Iteration 1:   log likelihood =  54.327539

Computing standard errors:

Mixed-effects ML regression                     Number of obs     =        117
Group variable: patient_id                      Number of groups  =         35

                                                Obs per group:
                                                              min =          3
                                                              avg =        3.3
                                                              max =          5

                                                Wald chi2(5)      =      24.68
Log likelihood =  54.327539                     Prob > chi2       =     0.0002
```

logtyk	Coef.	Std. Err.	z	P>|z|	[95% Conf.	Interval]
RedMeat	-.0010457	.0007471	-1.40	0.162	-.00251	.0004185
Whole_grains	-.0010173	.0003671	-2.77	0.006	-.0017369	-.0002978
age	.0253053	.0083229	3.04	0.002	.0089927	.0416179
sex_intro	.2353543	.146858	1.60	0.109	-.0524822	.5231908
bmi	-.0483713	.0214651	-2.25	0.024	-.090442	-.0063005
_cons	2.489547	.8572244	2.90	0.004	.8094184	4.169676

Random-effects Parameters	Estimate	Std. Err.	[95% Conf.	Interval]
**patient_id**: Identity				
var(_cons)	.1258377	.0305709	.0781665	.2025821
var(Residual)	.0066934	.0010453	.0049285	.0090904

LR test vs. linear model: chibar2(01) = **203.64** Prob >= chibar2 = **0.0000**

```
. mixed logtyk RedMeat Whole_grains bmi ||patient_id:

Performing EM optimization:

Performing gradient-based optimization:

Iteration 0:   log likelihood =  50.209524
Iteration 1:   log likelihood =  50.209524

Computing standard errors:

Mixed-effects ML regression                     Number of obs     =        117
Group variable: patient_id                      Number of groups  =         35

                                                Obs per group:
                                                              min =          3
                                                              avg =        3.3
                                                              max =          5

                                                Wald chi2(3)      =      12.15
Log likelihood =  50.209524                     Prob > chi2       =     0.0069
```

logtyk	Coef.	Std. Err.	z	P>|z|	[95% Conf.	Interval]
RedMeat	-.0011363	.000803	-1.41	0.157	-.0027101	.0004376
Whole_grains	-.0010952	.0004113	-2.66	0.008	-.0019013	-.000289
bmi	-.0419001	.0238749	-1.75	0.079	-.0886941	.0048939
_cons	4.326658	.6762727	6.40	0.000	3.001188	5.652128

Random-effects Parameters	Estimate	Std. Err.	[95% Conf.	Interval]
**patient_id**: Identity				
var(_cons)	.1598137	.0386807	.0994472	.256824
var(Residual)	.0066926	.0010451	.0049281	.0090889

LR test vs. linear model: chibar2(01) = **225.23** Prob >= chibar2 = **0.0000**

```
.
end of do-file

.
```
