# Supplementary material for: The Relation between Red Meat and Whole-Grain Intake and the Colonic Mucosal Barrier: A Cross-Sectional Study
Source: Nutrients. 2020 Jun 12;12(6):1765. doi: 10.3390/nu12061765 (PMC7353246; doi:10.3390/nu12061765)
Supplement: Supplementary file 1 [file nutrients-12-01765-s001.zip › Supplementary materials/Stata output3.pdf]

```
. do "C:\Users\pek3at\AppData\Local\Temp\STD1a44_000000.tmp"

. replace Whole_grains = . if energi<1600 | energi>30000
(3 real changes made, 3 to missing)

. replace RedMeat  = . if energi<1600 | energi>30000
(3 real changes made, 3 to missing)

.
. meglm RedMeat Epcam ||kost:, family(gamma) eform

Fitting fixed-effects model:

Iteration 0:   log likelihood = -843.00171
Iteration 1:   log likelihood = -797.56923
Iteration 2:   log likelihood = -795.15535
Iteration 3:   log likelihood = -795.05522
Iteration 4:   log likelihood = -795.05509
Iteration 5:   log likelihood = -795.05509

Refining starting values:

Grid node 0:   log likelihood = -846.09321

Fitting full model:

Iteration 0:   log likelihood = -846.09321  (not concave)
Iteration 1:   log likelihood = -804.74705
Iteration 2:   log likelihood = -793.66871
Iteration 3:   log likelihood = -792.88907
Iteration 4:   log likelihood = -792.84902
Iteration 5:   log likelihood = -792.84854
Iteration 6:   log likelihood = -792.84854

Mixed-effects GLM                               Number of obs     =        154
Family:                    gamma
Link:                        log
Group variable:             kost                Number of groups  =        154

                                                Obs per group:
                                                              min =          1
                                                              avg =        1.0
                                                              max =          1

Integration method: mvaghermite                 Integration pts.  =          7

                                                Wald chi2(1)      =       1.17
Log likelihood = -792.84854                     Prob > chi2       =     0.2804
------------------------------------------------------------------------------
     RedMeat |     exp(b)   Std. Err.      z    P>|z|     [95% Conf. Interval]
-------------+----------------------------------------------------------------
       Epcam |   1.087286    .084292     1.08   0.280     .9340155    1.265707
       _cons |   74.95681   6.494386    49.82   0.000     63.25015    88.83021
-------------+----------------------------------------------------------------
       /logs |  -.9037341   .2593355                     -1.412022   -.3954459
-------------+----------------------------------------------------------------
kost         |
   var(_cons)|   .1328004    .095428                      .0324744    .5430726
------------------------------------------------------------------------------
Note: Estimates are transformed only in the first equation.
LR test vs. gamma model: chibar2(01) = 4.41           Prob >= chibar2 = 0.0178
```

```
. meglm Whole_grains Epcam ||kost:, family(gamma) eform

Fitting fixed-effects model:

Iteration 0:   log likelihood = -936.11017
Iteration 1:   log likelihood = -923.95834
Iteration 2:   log likelihood = -903.04589
Iteration 3:   log likelihood = -903.03303
Iteration 4:   log likelihood = -903.03303

Refining starting values:

Grid node 0:   log likelihood =  -945.2048

Fitting full model:

Iteration 0:   log likelihood =  -945.2048  (not concave)
Iteration 1:   log likelihood = -903.38032
Iteration 2:   log likelihood = -903.02462
Iteration 3:   log likelihood = -903.02324
Iteration 4:   log likelihood = -903.02323

Mixed-effects GLM                               Number of obs     =        154
Family:                       gamma
Link:                           log
Group variable:                kost             Number of groups  =        154

                                                Obs per group:
                                                              min =          1
                                                              avg =        1.0
                                                              max =          1

Integration method: mvaghermite                 Integration pts.  =          7

                                                Wald chi2(1)      =       0.53
Log likelihood = -903.02323                     Prob > chi2       =     0.4677
------------------------------------------------------------------------------
Whole_grains |     exp(b)   Std. Err.      z    P>|z|     [95% Conf. Interval]
-------------+----------------------------------------------------------------
       Epcam |   1.060185   .0853185     0.73   0.468     .9054842    1.241315
       _cons |   149.8561   14.50016    51.77   0.000     123.9686    181.1495
-------------+----------------------------------------------------------------
       /logs |  -.4954618   .0750115                     -.6424816    -.348442
-------------+----------------------------------------------------------------
kost         |
   var(_cons)|   .0060186   .0437974                      3.85e-09     9412.58
------------------------------------------------------------------------------
Note: Estimates are transformed only in the first equation.
LR test vs. gamma model: chibar2(01) = 0.02           Prob >= chibar2 = 0.4443

.
end of do-file

.
```
