# Supplementary material for: The Relation between Red Meat and Whole-Grain Intake and the Colonic Mucosal Barrier: A Cross-Sectional Study
Source: Nutrients. 2020 Jun 12;12(6):1765. doi: 10.3390/nu12061765 (PMC7353246; doi:10.3390/nu12061765)
Supplement: Supplementary file 1 [file nutrients-12-01765-s001.zip › Supplementary materials/Supplementary table S1.pdf]

Table S1: The mental and physical health-related quality of life data of the included subjects using SF-12 questionnaire

| | Component t-score | Median | IQR | t-score < mean[1] |
|---|---|---|---|---|
| **Physical** | NEMC physical functioning | 56.47 | (47.88, 56.47) | 67 (42.1%) |
| | NEMC role limitation physical t-score | 52.57 | (43.36, 57.17) | 76 (47.8%) |
| | NEMC pain t-score | 57.44 | (47.25, 57.44) | 75 (47.2%) |
| | NEMC general health t-score | 44.74 | (44.74, 55.52) | 102 (64.2%) |
| | **NEMC physical health t-score - sf12** | 51.02 | (43.24, 55.09) | 76 (47.8%) |
| **Mental** | NEMC vitality t-score | 57.81 | (47.75, 57.81) | 73 (45.9%) |
| | NEMC social functioning t-score | 56.57 | (46.47, 56.57) | 41 (25.8%) |
| | NEMC role limitation emotional | 56.09 | (44.90, 56.09) | 53 (33.3%) |
| | NEMC mental health t-score | 52.35 | (46.25, 58.44) | 46 (28.9%) |
| | **NEMC mental health t-score - sf12** | 54.20 | (47.21, 58.15) | 52 (32.7%) |

[1]The number and the percentage of subjects with a t-score< 50. The values are rounded to second decimal places (hundredths). Abbreviations: SF-12, the short form 12 health survey; NEMC, New England Medical Center.
